# Population structure and interspecific hybridisation of two invasive blowflies (Diptera: Calliphoridae) following replicated incursions into New Zealand

**DOI:** 10.1002/ece3.10832

**Published:** 2024-01-07

**Authors:** Lilly Croft, Paige Matheson, Chloe Flemming, Nathan J. Butterworth, Angela McGaughran

**Affiliations:** ^1^ Te Aka Mātuatua – School of Science University of Waikato Hamilton New Zealand; ^2^ School of Biological Sciences Monash University Melbourne Victoria Australia

**Keywords:** biological invasion, blowflies, gene flow, hybridisation, invertebrates, population genomics

## Abstract

Rates of biological invasion are increasing globally, with associated negative effects on native biodiversity and ecosystem services. Among other genetic processes, hybridisation can facilitate invasion by producing new combinations of genetic variation that increase adaptive potential and associated population fitness. Yet the role of hybridisation (and resulting gene flow) in biological invasion in invertebrate species is under‐studied. *Calliphora hilli* and *Calliphora stygia* are blowflies proposed to have invaded New Zealand separately from Australia between 1779 and 1841, and are now widespread throughout the country. Here, we analysed genome‐wide single nucleotide polymorphisms (SNPs), generating genotyping‐by‐sequencing data for 154 individuals collected from 24 populations across New Zealand and Australia to assess the extent of gene flow and hybridisation occurring within and between these blowflies and to better understand their overall population structure. We found that New Zealand populations of both species had weak genetic structure, suggesting high gene flow and an absence of dispersal limitations across the country. We also found evidence that interspecific hybridisation is occurring in the wild between *C*. *hilli* and *C*. *stygia* in both the native and invasive ranges, and that intraspecific admixture is occurring among populations at appreciable rates. Collectively, these findings provide new insights into the population structure of these two invasive invertebrates and highlight the potential importance of hybridisation and gene flow in biological invasion.

## Graphical abstract

Rates of biological invasion are increasing globally, with associated negative effects on native biodiversity and ecosystem services. Yet the role of hybridisation (and resulting gene flow) in biological invasion in invertebrate species is under‐studied. We analysed genome‐wide single nucleotide polymorphism (SNP) data for two blowfly species collected from 24 populations across New Zealand and Australia to provide new insights into the population structure of these two invasive invertebrates and highlight the potential importance of hybridisation and gene flow in biological invasion.

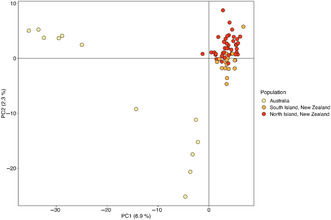

## 1 INTRODUCTION

Invasive species—populations of organisms that are self‐sustaining with evidence of spread from the original geographic location (Hobbs, 2000)—are one of the biggest threats to biodiversity, natural and cultivated ecosystems, primary industries and the economy (Keller & Taylor, 2010). Rates of invasions have increased substantially in recent decades as climate change and other anthropogenic impacts redefine species ranges across the globe (Seebens et al., 2017, 2021; Spence & Tingley, 2020). Despite this, little is known about the mechanisms through which species can overcome certain evolutionary challenges associated with the invasion process (e.g. genetic bottlenecks and changes in selection pressures) that would normally constrain their introduction, establishment and spread.

Two processes that are hypothesised to promote invasion success are hybridisation and associated gene flow. Hybridisation occurs when individuals from genetically differentiated populations (including different species) reproduce to create offspring with mixed genetic characteristics (Abbott et al., 2013), while gene flow more broadly relates to the transfer of genetic material between populations. Both can act together to increase adaptive potential and/or population fitness in a variety of ways—for example, alleviating the loss of genetic diversity often experienced during colonisation (e.g. due to the establishment of a population from a small pool of founders), generating novel genotypes, promoting hybrid vigour (i.e. the enhanced fitness of the hybrid relative to either parent; Ackermann, 2010; Keller & Taylor, 2010; Qiao et al., 2019; Rius & Darling, 2014) through genetic admixture (i.e. the incorporation of new alleles into existing lineages), masking or unloading deleterious recessive alleles and/or transferring beneficial alleles from locally adapted to invasive species (or vice versa; Ackermann, 2010; Keller & Taylor, 2010; Lee, 2002; Qiao et al., 2019; Rius & Darling, 2014).

Numerous studies have documented the positive effects of hybridisation and gene flow on invasion success in a number of taxa. For example—in the plant genus *Tamarix* (Caryophyllales: Tamaricaceae), *T*. *e*. *ramosissima* and *T*. *chinensis* hybrids account for up to 87% of the invasive population in Asia (Gaskin, 2017); in fish, early generation hybridisation between two invasive carp species (silver carp: *Hypophthalmichthys molitrix* and bighead carp: *Hypophthalmichthys nobilis*; both Cypriniformes: Cyprinidae) has been shown to facilitate rapid adaptation and range expansion in the Illinois River, USA (Coulter et al., 2019); in mammals, directional hybridisation and introgression between the European brown hare (*Lepus europaeus*) and the endemic Irish hare (*L*. *timidus hibernicus*; both Lagomorpha: Leporidae) favours the invasive species over the endemic (Reid et al., 2022). The latter example may have implications for the endemic hare's long‐term survival and highlights that hybridisation can also act maladaptively during invasions by creating hybrids with lower fitness than their parents or with an inability to produce viable offspring (Hoskin & Higgie, 2013).

Despite these findings across a range of species, the extent to which hybridisation and gene flow occurs in invertebrates (and its prevalence during or post‐invasion) has received little attention in the literature (Kirk et al., 2013)—though one notable study found that hybridisation between the invasive pest moth *Helicoverpa armigera* and a local species (*Helicoverpa zea*) in Brazil rapidly transferred genes that conferred resistance to fenvalerate (a broad spectrum pesticide), resulting in hybrids that were both highly adapted to local conditions and resistant to insecticides (Pearce et al., 2017; Valencia‐Montoya et al., 2020).

Blowflies—like many invertebrates—are often successfully invasive due to their rapid generation times, high fecundity and ability to inconspicuously stow away on transport cargo, such as shipping containers, and be subsequently transported long distances. Two widespread invasive blowfly species in New Zealand are *Calliphora hilli* and *Calliphora stygia*, which were likely introduced from Australia (the only other country they are known from) between 1779 and 1841 (Dear, 1986). Both species are very similar morphologically, fulfil the same ecological niche (Dear, 1986; Muller, 1939) and cause primary (*C*. *stygia*) and secondary (*C*. *hilli*) flystrike—an ectoparasitic disease that occurs when female blowflies deposit eggs onto a host's skin (commonly sheep in New Zealand) and resulting larvae hatch and feed on the living host tissue (Heath & Bishop, 2006). As well as potentially being important agricultural pests, blowflies fulfil important ecosystem services as pollinators, nutrient recyclers and decomposers (Arias‐Robledo et al., 2019). Yet we understand little about their population structure and the extent to which genetic processes, such as hybridisation and gene flow, operate during or after invasion.

Such questions are particularly interesting for blowflies, given that they are highly vagile, excellent dispersers (Butterworth et al., 2023; Tsuda et al., 2009) that may be expected to maintain high rates of population connectivity post‐invasion. Furthermore, research under laboratory conditions has demonstrated that *C*. *stygia* can produce hybrids with *C*. *albifrontalis* in Australia and *C*. *hilli* can produce hybrids with *C*. *varifrons*—suggesting that hybridisation within the species complex may be occurring in the wild (Monzu, 1977; Wallman & Adams, 1997), though this has not yet been confirmed using genomic data. Here, we analyse genome‐wide single nucleotide polymorphisms (SNPs) from samples collected from various locations across New Zealand and Australia to investigate population structure, hybridisation and gene flow (both within and between wild *C*. *hilli* and *C*. *stygia*)—of these co‐invading species.

## 2 MATERIALS AND METHODS

### 2.1 Sample collection and identification

Sampling kits and set‐up instructions were sent to friends and colleagues to use in their backyards in various locations across New Zealand (Table 1). Sampling traps consisted of a modified bottle trap (Hwang & Turner, 2005) made from two plastic bottles, with meat bait placed in the lower bottle, which was covered in black tape to block out light. Flies could enter the trap through slots in the side of the lower bottle and were funnelled towards the light in the upper bottle, where they were trapped until collection. Traps were left outside for 3–4 days and were checked and emptied daily. Emptying traps involved placing the upper bottle in the freezer to euthanise the flies, which were then placed into a 50‐mL falcon tube containing 69% ethanol for postage back to the University of Waikato.

**TABLE 1:** Sampling information for *Calliphora hilli* and *Calliphora stygia*, including population names, population codes, GPS coordinates, population sample numbers and observed (*H*_o_) and expected (*H*_e_) heterozygosity (based on the neutral data set).

Population name	Population code	GPS coordinates	No. samples		Heterozygosity			
			*C*. *hilli*	*C*. *stygia*	*C*. *hilli*—*H*_o_	*C*. *hilli*—*H*_e_	*C*. *stygia*—*H*_o_	*C*. *stygia*—*H*_e_
Kerikeri	KRI	−35.2089, 173.9619	5	3	0.11	0.25	0.10	0.27
Karangahake	KGK	−37.4343, 175.7255	5	–	0.10	0.25	–	–
Te Aroha	TEA	−37.5386, 175.6932	5	5	0.08	0.24	0.10	0.28
Kaniwhaniwha	PKW	−37.9339, 175.0777	5	5	0.08	0.25	0.09	0.27
Pirongia	PGR	−37.9683, 175.1504	–	5	–	–	0.10	0.28
Tauranga	TGA	−37.7327, 176.1799	5	–	0.12	0.25	–	–
Taranaki Ōakura	TAO	−39.1157, 173.9522	4	5	0.09	0.24	0.10	0.27
Palmerston North	PMN	−40.3785, 175.5866	5	4	0.10	0.24	0.12	0.26
Wellington	WLG	−41.2950, 174.7989	5	5	0.11	0.24	0.11	0.26
Wellington Te Papa	WTP	−41.2904, 174.7820	–	3	–	–	0.12	0.25
Blenheim	BHE	−41.5075, 173.9299	5	4	0.09	0.24	0.11	0.27
Marlborough	MLB	−41.9805, 173.6659	–	3	–	–	0.12	0.28
Greymouth	GMN	−42.4646, 171.2029	5	5	0.08	0.24	0.10	0.27
Christchurch	CHC	−43.5317, 172.5794	–	5	–	–	0.10	0.27
Dunedin Fairfield	DUF	−45.9000, 170.3823	5	4	0.09	0.24	0.11	0.26
Dunedin Ravensbourne	DUR	−45.8640, 170.5494	5	4	0.14	0.25	0.10	0.32
Jervis Bay, NSW, Australia	JBA	−35.0925, 150.6187	5	–	0.07	0.21	–	–
Mt Keira, NSW, Australia	MNA	−34.3970, 150.8534	3	–	0.07	0.15	–	–
Blackheath, NSW, Australia	BNA	−33.6144, 150.2683	4	4	0.07	0.20	0.11	0.26
Echo Point, NSW, Australia	ENA	−33.7296, 150.3116	–	5	–	–	0.09	0.26
Seaford, NSW, Australia	SVA	−33.7928, 151.2409	–	3	–	–	0.11	0.25
Canberra, ACT, Australia	CAA	−35.2803, 149.1310	–	3	–	–	0.11	0.24
Mt Crawford, SA, Australia	MSA	−34.7185, 138.9579	–	4	–	–	0.11	0.26
Hobart, TAS, Australia	HTA	−42.8829, 147.3264	–	4	–	–	0.10	0.27

*Note*: New Zealand populations are listed in rough geographical order from the top of the North Island to the bottom of the South Island. See Table S1 for further sampling details.

**FIGURE 1:**
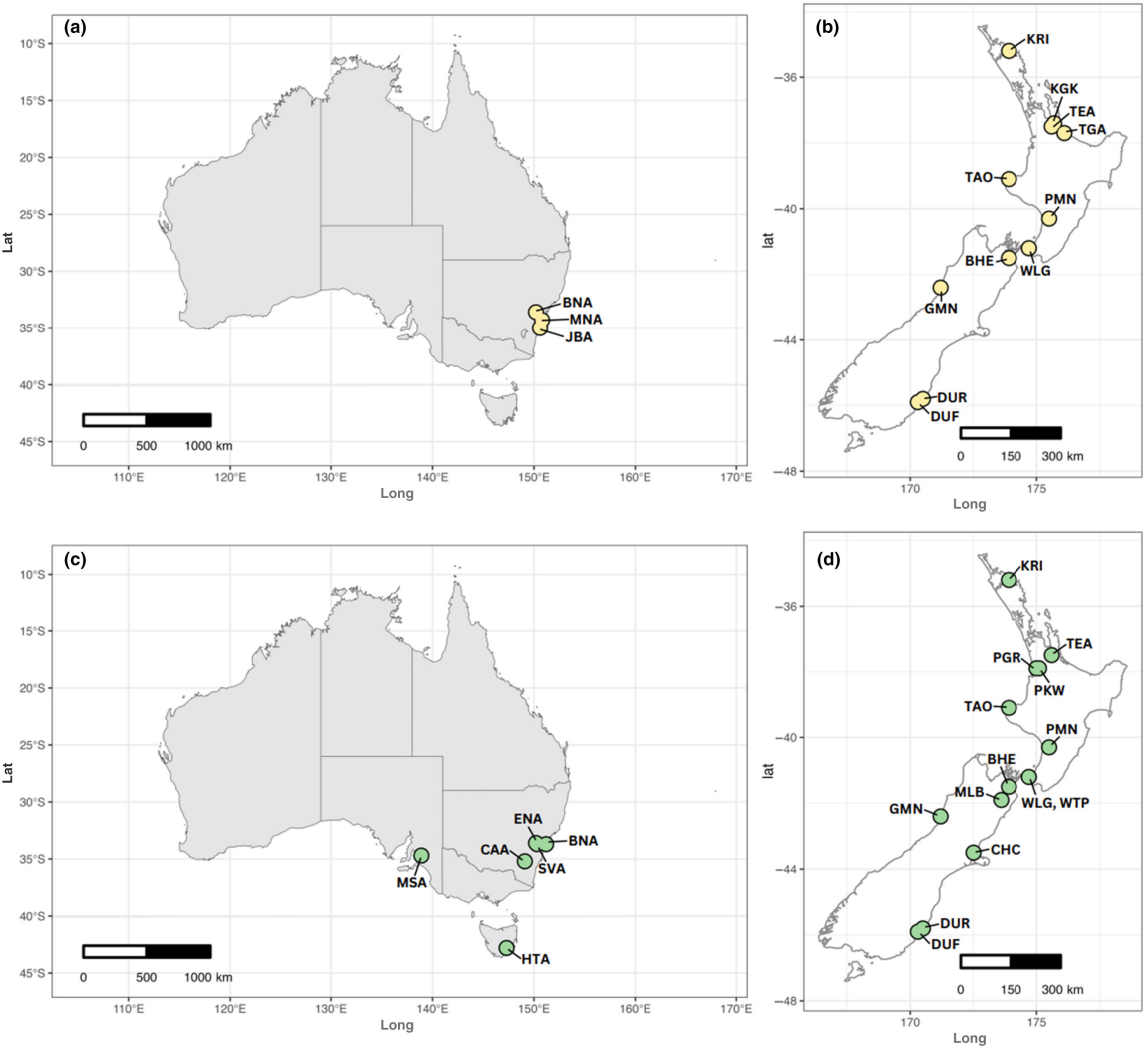
Geographical maps showing sampling sites where *Calliphora hilli* (yellow dots) and *Calliphora stygia* (green dots) were collected in Australia (left) and New Zealand (right). Refer to Table 1 for location codes.

Alongside the New Zealand backyard sites, we obtained 32 samples from eight locations in Australia. Together, this resulted in a total of 75 samples from 16 sites for *C*. *hilli* and 89 samples from 21 sites for *C*. *stygia* (Figure 1; Table 1). All specimens were identified to species level using the taxonomic key of Dear (1986).

### 2.2 DNA extraction and sequencing

DNA was extracted for 162 samples using a DNeasy Blood & Tissue kit (Qiagen) and associated protocol, quantified using a Qubit fluorometer (Thermo Fisher Scientific) and sent to AgResearch Ltd for genotyping‐by‐sequencing (GBS).

A single GBS library was constructed according to the methods outlined in Elshire et al. (2011), with modifications as outlined in Dodds et al. (2015). The GBS library was prepared using a PstI‐MspI double‐digest and included negative control samples (no DNA). Libraries underwent a Pippin Prep (SAGE Science, Beverly, Massachusetts, United States) to select fragments in the size range of 220–340 bp (genomic sequence plus 148 bp of adapters). Single‐end sequencing (1 × 101 bp) was performed on a NovaSeq6000 utilising v1.5 chemistry.

### 2.3 SNP filtering

Raw fastq files were quality checked using FastQC v0.10.1 (http://www.bioinformatics.babraham.ac.uk/projects/fastqc/). IPYRAD v0.7.28 (Eaton & Overcast, 2020) was used to filter and remove low quality data, identify homology among reads through de novo assembly (i.e. no reference genome used), make SNP calls and format output files for each species data set individually. Reads were processed with the following non‐default parameter settings: filter_adapters (2, where adapters were removed), filter_min_trim_len (60) and trim_reads (10, −140, 0, 0); and SNPs were exported in variant call format (VCF).

The VCF file was filtered using VCFTOOLS v0.1.13 (Danecek et al., 2011), with ‐‐missing‐indv, ‐‐max‐missing‐count and ‐‐maf parameters applied to filter data with >98% missing data, 20% missing genotypes across all individuals, and a minor allele frequency cut‐off of 5%. This resulted in data sets of 16,144 and 12,494 SNPs, for *C*. *hilli* and *C*. *stygia*, respectively.

Because of the morphological similarities between *C*. *stygia* and *C*. *hilli*, a combined data set (i.e. fastq files for both species were processed together) was also run through the IPYRAD and VCFTOOLS pipeline outlined above, and an initial principal component analysis (PCA; see below) of this combined data set (16,333 SNPs) was performed. Any potential hybrids (i.e. samples that overlapped in the PC space) were then removed from the individual species data sets to create ‘pure’ VCF files for population analysis for each species (see Section 3; Table S1).

### 2.4 Kinship analysis

To assess relatedness of individuals prior to genetic analysis, a kinship analysis was performed using the ‐‐relatedness2 function implemented in VCFTOOLS. This function produces pairwise kinship coefficients of individuals based on the algorithm described by Manichaikul et al. (2010), where relatedness is calculated based on the probability of finding identical alleles when randomly sampling one allele from each heterozygous individual. We took a conservative approach, removing six individuals that had relatedness factors >0.01 to prevent any potential artificial inflation of population structure results. This also resulted in the removal of two further individuals from the HYT population, due to the sample size of that population being reduced to *n* < 3. Thus, the final data set for population analysis contained 71 samples from 15 sites for *C*. *hilli* and 83 samples from 20 sites for *C*. *stygia* (Figure 1, Table 1 and Table S1).

### 2.5 Population analyses

The following analyses were conducted in R v4.3.0 (R Core Team, 2020). Geographical maps were first created to visualise the geographical distribution of samples using the function ‘map_data’ within the ggplot2 package v3.3.6; (Wickham, 2016). Genetic diversity (heterozygosity) and pairwise differentiation (*F*_ST_) were determined for each population and species using the hierfstat package v0.5‐11 (Goudet, 2005) on the neutral data set.

PCAs were conducted using the ‘glPCA’ function implemented in the adegenet package v2.1.10 (Jombart, 2008) and plotted using ggplot2. Admixture analyses were conducted by first converting the VCF file into geno format using the R package, LEA v3.6.0 (Frichot & François, 2015). The optimal K value was determined using a cross entropy plot produced by using the ‘snmf’ function in LEA on the geno file. The function ‘qmatrix’ from the tess3r package v1.1.0 (Caye & Francois, 2016), along with ggplot, was used to produce an admixture bar plot for each species. Using the ‘meta’ function within the terra package (Hijmans, 2023), a new VCF file containing only neutral SNPs was created, and both the PCA and admixture analyses were repeated on these neutral data sets. The neutral and non‐neutral data sets produced consistent results; thus, we present the neutral plots in the main text and provide the non‐neutral plots in the Supporting Information.

Finally, we investigated potential hybrids between *C*. *stygia* and *C*. *hilli* in several ways: (i) we examined the PCA for the combined species data set (see above); (ii) we performed admixture analysis for *K* = 2–5 as outlined above on the combined data set; and (iii) we performed a specific hybrid analysis using the ‘gl.nhybrids’ function from the DartR package v2 (Gruber et al., 2018; Mijangos et al., 2022). The ‘gl.nhybrids’ function creates an input file for the programme, NewHybrids (Anderson, 2003), and executes it in order to assign samples to categories of either parental population (in this case, *C*. *hilli* or *C*. *stygia*), *F*_*1*_ or *F*_*2*_ hybrids, or backcrosses to either of the parental species. Because only 200 random loci can be retained for the NewHybrids analysis, we repeated the analysis 10 times (using the ‘random’ method), with resultant posterior probabilities combined and averaged (Table S2). Based on these results, individuals were identified as ‘pure’ species, hybrids or backcrosses. We subsequently plotted results in a bar chart, as per Baiakhmetov et al. (2021). A similar protocol was followed by Hill et al. (2022) to identify hybrids of sambar deer (*Cervus unicolor*) and rusa deer (*Cervus timorensis*) using GBS data.

## 3 RESULTS

### 3.1 Calliphora hilli

Observed heterozygosity (*H*_o_) was low across all 15 *C*. *stygia* populations and was significantly lower than expected heterozygosity (*H*_e_) (mean *H*_o_ = 0.09, range = 0.07–0.14; mean *H*_e_ = 0.22, range = 0.15–0.250; T_28_ = −15.943; *p* < .001; Table 1).

We observed an overall lack of genetic structuring between *C*. *stygia* populations, with pairwise *F*_ST_ ranging from 0.00 (e.g. between Kerikeri and Pirongia Kaniwhaniwha) to 0.256 (Dunedin Ravensbourne vs. Mount Kiera). Within the islands of New Zealand, mean *F*_ST_ was 0.009 (North Island) and 0.021 (South Island), while mean *F*_ST_ between all populations in the North Island versus all populations in the South Island was 0.024. Mean *F*_ST_ between New Zealand and Australian populations was fourfold higher (0.076) and ranged from 0.000 to 0.207 within Australian populations (Table 2), although these values are potentially impacted by the low sample sizes of the Australian populations.

**TABLE 2:** *Calliphora hilli F*_ST_ values presented by population (population codes as referred to in Table 1) based on the neutral data set.

	KRI	KGK	TEA	PKW	TGA	TAO	PMN	WLG	BHE	GMN	DUF	DUR	JBA	MNA
KGK	0.004													
TEA	0.015	0.008												
PKW	0.000	0.000	0.016											
TGA	0.012	0.002	0.006	0.013										
TAO	0.004	0.000	0.025	0.002	0.016									
PMN	0.025	0.006	0.020	0.000	0.002	0.024								
WLG	0.024	0.000	0.025	0.000	0.000	0.008	0.000							
BHE	0.007	0.014	0.033	0.000	0.028	0.001	0.018	0.007						
GMN	0.045	0.039	0.014	0.015	0.056	0.026	0.032	0.031	0.018					
DUF	0.027	0.002	0.029	0.026	0.046	0.014	0.039	0.035	0.000	0.039				
DUR	0.027	0.014	0.054	0.004	0.037	0.019	0.027	0.015	0.024	0.021	0.022			
JBA	0.168	0.190	0.199	0.194	0.213	0.201	0.215	0.208	0.188	0.209	0.207	0.225		
MNA	0.211	0.220	0.233	0.204	0.230	0.221	0.231	0.240	0.245	0.254	0.207	0.256	0.000	
BNA	0.101	0.097	0.114	0.0.92	0.117	0.114	0.122	0.107	0.096	0.111	0.124	0.136	0.135	0.207

*Note*: New Zealand populations are listed in rough geographical order from the top of the North Island to the bottom of the South Island.

**FIGURE 2:**
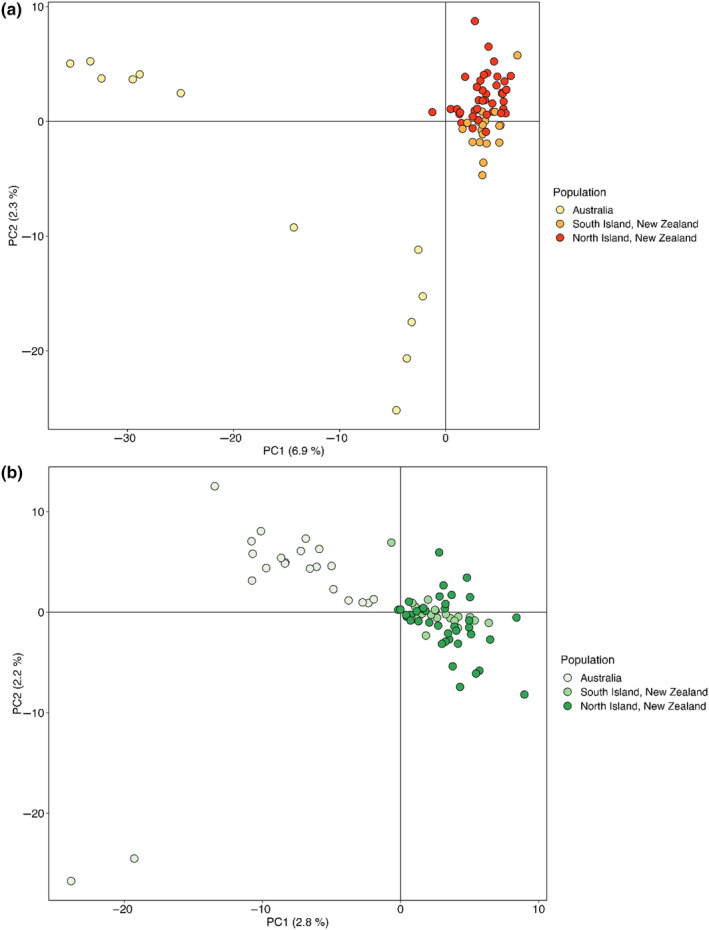
PCA plots showing: (a) *Calliphora hilli* samples (neutral data set: 16,636 SNPs) and (b) *Calliphora stygia* samples from the neutral data set (12,841 SNPs).

These findings were reinforced by the PCA, in which individuals from Australia formed two main clusters distinct from New Zealand and there was no clear separation between the North and South Islands (Figure 2a and Figure S1a). The Australian samples in the upper cluster corresponded to Mt Keira (MNA) and Jervis Bay (JBA), while the lower cluster of individuals corresponded to NSW (Blackheath/BNA and Jervis Bay) and a more centrally located individual between the two Australian groups was from Jervis Bay (Figure S2b).

**FIGURE 3:**
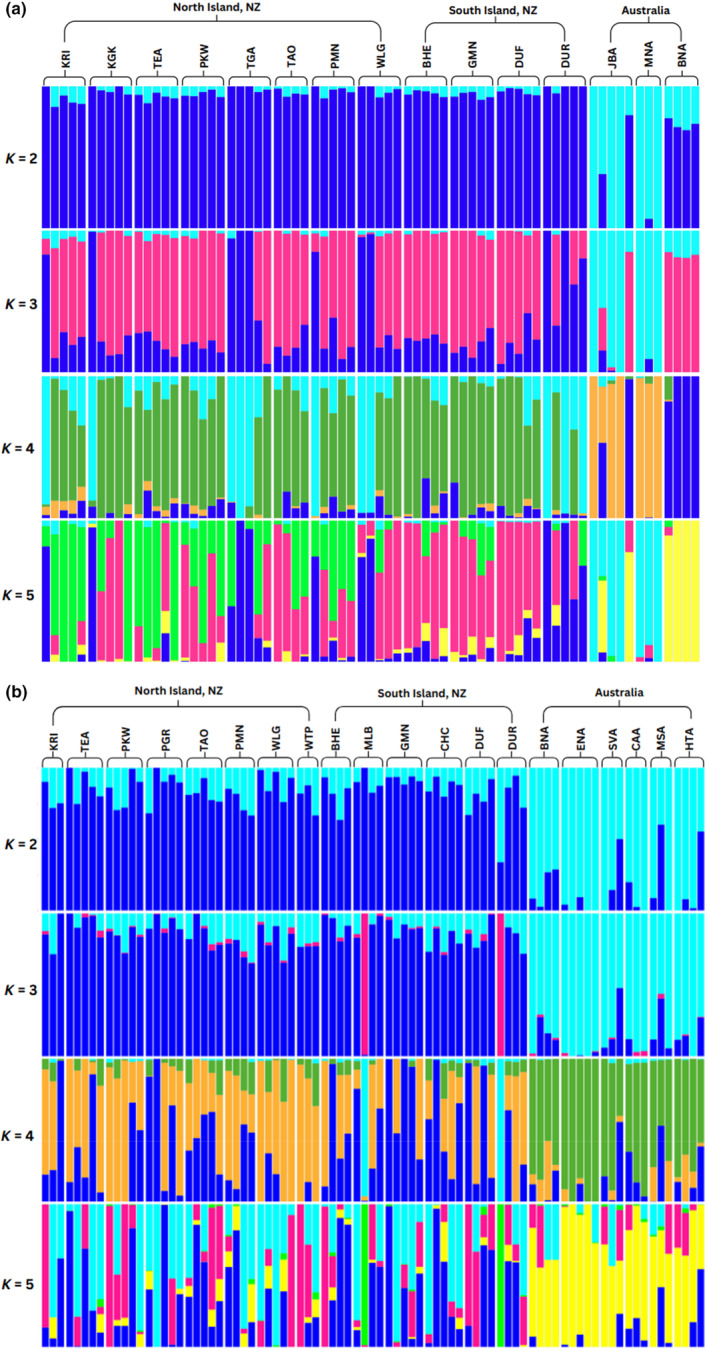
Admixture plots for: (a) *Calliphora hilli* (neutral data set: 16,636 SNPs; optimal *K* = 2) and (b) *Calliphora stygia* (neutral data set: 12,841 SNPs; optimal *K* = 2) produced using sparse non‐negative matrix factorisation (sNMF). Admixture proportions showing *K* = 2–5 are presented, with populations in order from left to right corresponding to the top of the North Island, through to the bottom of the South Island of New Zealand, followed by Australia.

Sparse non‐negative matrix factorisation (sNMF) analysis of the neutral data set indicated an optimal *K*‐value of two clusters, which largely corresponded to New Zealand vs. Australia. At *K* = 2, New Zealand populations of *C*. *hilli* showed no clear difference in admixture patterns between the North and South Islands and a small degree of shared ancestry/admixture (<10%) with Australia. At *K* = 3, a third genetic group was introduced, which was mostly present in New Zealand and increased the degree of admixture there. This trend was continued at *K*‐values of four and five, where the Australian populations also separated off as more distinct genetic groups (JBA + MNA, and BNA) with a small number of linkages to New Zealand via individuals from JBA (Figure 3a and Figure S2a).

### 3.2 Calliphora stygia

*H*_o_ was low within all 20 *C*. *stygia* populations and was significantly lower than *H*_e_ (mean *H*_o_ = 0.11, range = 0.09–0.12; mean *H*_e_ = 0.27, range = 0.24–0.32; *T*_38_ = −39.26; *p* < .001; Table 1).

Similar to the *C*. *hilli* results, *C*. *stygia* demonstrated an overall lack of genetic structuring between populations, with pairwise *F*_ST_ ranging from 0.000 (numerous examples) to 0.096 (DUR vs. ENA) (Table 3). Within New Zealand's North and South Islands, mean *F*_ST_ was low (North: 0.005, South: 0.009), while mean *F*_ST_ between all populations in the North Island versus all populations in the South Island was 0.008. Australia and New Zealand showed approximately two times higher genetic differentiation, with a mean *F*_ST_ of 0.026, and *F*_ST_ among Australian populations was relatively low (maximum: 0.045; Table 3).

**TABLE 3:** *Calliphora stygia F*_ST_ values presented by population (population codes as referred to in Table 1) based on the neutral data set.

	KRI	TEA	PKW	PGR	TAO	PMN	WLG	WTP	BHE	MLB	GMN	CHC	DUF	DUR	BNA	ENA	SVA	CAA	MSA
TEA	0.000																		
PKW	0.000	0.000																	
PGR	0.001	0.000	0.018																
TAO	0.000	0.000	0.000	0.000															
PMN	0.004	0.000	0.000	0.004	0.011														
WLG	0.003	0.000	0.000	0.001	0.001	0.000													
WTP	0.033	0.020	0.000	0.007	0.009	0.025	0.013												
BHE	0.000	0.000	0.000	0.000	0.000	0.000	0.000	0.013											
MLB	0.000	0.014	0.000	0.003	0.014	0.003	0.008	0.022	0.000										
GMN	0.004	0.006	0.000	0.009	0.017	0.025	0.027	0.003	0.000	0.016									
CHC	0.000	0.000	0.015	0.003	0.001	0.000	0.015	0.017	0.000	0.026	0.005								
DUF	0.010	0.002	0.001	0.008	0.019	0.016	0.032	0.027	0.000	0.000	0.000	0.015							
DUR	0.011	0.006	0.004	0.022	0.013	0.000	0.000	0.000	0.000	0.000	0.033	0.0026	0.008						
BNA	0.051	0.052	0.038	0.046	0.060	0.035	0.040	0.038	0.030	0.035	0.068	0.052	0.028	0.041					
ENA	0.083	0.079	0.076	0.079	0.082	0.055	0.082	0.058	0.063	0.069	0.092	0.090	0.095	0.096	0.033				
SVA	0.028	0.036	0.030	0.024	0.028	0.021	0.057	0.043	0.039	0.040	0.032	0.032	0.057	0.000	0.022	0.033			
CAA	0.060	0.060	0.061	0.062	0.075	0.042	0.078	0.070	0.060	0.066	0.058	0.062	0.058	0.048	0.020	0.045	0.007		
MSA	0.021	0.041	0.023	0.003	0.039	0.012	0.045	0.032	0.017	0.038	0.033	0.045	0.048	0.018	0.000	0.018	0.012	0.011	
HTA	0.034	0.049	0.035	0.047	0.049	0.032	0.054	0.031	0.036	0.042	0.063	0.034	0.053	0.015	0.029	0.030	0.000	0.001	0.00

*Note*: New Zealand populations are listed in rough geographical order from the top of the North Island to the bottom of the South Island.

We found no clear signal of geographical structure within New Zealand's North and South islands for *C*. *stygia* based on the PCA, reinforcing our findings from *F*_ST_ (Figure 2a and Figure S1c,d). Furthermore, *C*. *stygia* displayed less separation between New Zealand and Australian samples, with the first two principal components explaining only 5% of the total genetic variance (Figure 2b) compared to *C*. *hilli*, where they explained 9.2% (Figure 2a).

Similar to the *C*. *hilli* admixture results, at *K* = 2 (the optimal *K*‐value), both New Zealand and Australian populations demonstrated signals of admixture. Rates of admixture were relatively consistent within New Zealand populations, which again showed no clear distinction between North and South Islands, and all New Zealand populations shared genetic ancestry with the Australian group, though the degree of admixture varied among individuals (Figure 3b and Figure S2b). In contrast to *C*. *hilli*, at *K* = 3, the degree of admixture did not increase significantly; instead, two New Zealand individuals (from MLB and DUR) separated off and this new genetic cluster (pink colour in Figure 3b) showed up at very small (<5%) degrees of admixture in other New Zealand and Australian samples. At *K* = 4 and *K* = 5, Australian and New Zealand samples maintained their distinctness, while admixture rates increased across most samples in a non‐geographic manner (Figure 3b and Figure S2b).

### 3.3 Hybrid analyses

**FIGURE 4:**
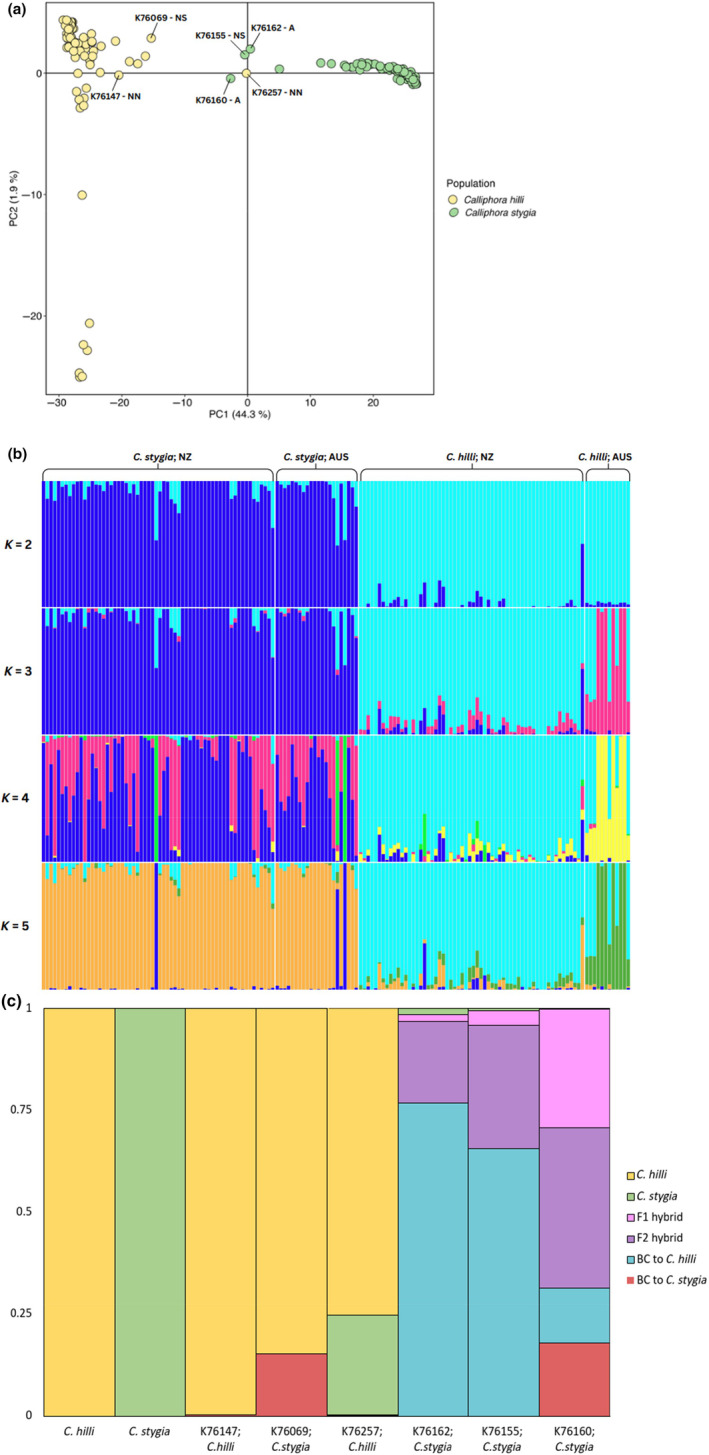
Hybrid analyses for *Calliphora hilli* and *Calliphora stygia* samples using the combined species neutral data set (12,155 SNPs). (a) PCA plot including potential hybrids identified by the NewHybrids analysis labelled by individual codes. Individuals are coloured by taxonomically identified species as per the key. ‘‐A’ in the individual code indicates Australian samples, ‘‐NN’ indicates New Zealand North Island and ‘‐NS’ indicates New Zealand South Island samples; (b) Admixture plot, produced using sparse non‐negative matrix factorisation (sNMF) analysis; and (c) NewHybrids analysis, indicating ‘pure’, hybrid or backcrossed status of six individuals using a threshold of 0.01—F1 and F2 hybrids represent offspring from first‐ and second‐generation crosses between *C*. *stygia* and *C*. *hilli*, respectively, BC to *C*. *hilli* and BC to *C*. *stygia* indicates first generation back‐crossed individuals to the respective species, each individual is labelled according to its taxonomic identification, with individual codes corresponding to Table S1.

Generally, *C*. *hilli* and *C*. *stygia* individuals clustered together within their ‘pure’ species group on the combined PCA; however, several individuals from both species can be observed together in the centre of the plot (Figure 4a and Figure S3a). Similarly, the combined species admixture plot (optimal *K* = 2) revealed two distinct genetic groups corresponding to each species, with some individuals displaying small degrees of admixture potentially suggestive of putative hybrids (Figure 4b and Figure S3b). Neither species demonstrated a clear differentiation between New Zealand and Australian individuals at *K* = 2, although higher values of *K* resulted in the differentiation of Australian and New Zealand samples for *C*. *hilli*.

Out of 154 *C*. *hilli* and *C*. *stygia* samples, the NewHybrids analysis identified five hybrid and/or backcrossed individuals, including two from Australia (Figure 4c and Figure S3c, Tables S2 and S3). The first two columns of the plot (Figure 4c) provide examples of ‘pure’ *C*. *hilli* and *C*. *stygia* individuals. Individual K76257 (*C*. *hilli*; Wellington, Te Papa) showed a hybrid genetic pattern, with 76% and 24% genetic contributions from *C*. *hilli* and *C*. *stygia*, respectively. Furthermore, individuals K76160 (*C*. *stygia*; Mt Crawford, Australia), K76155 (*C*. *stygia*; Marlborough, New Zealand) and K76162 (*C*. *stygia*; Mt Crawford, Australia) showed a hybrid/backcrossed genetic pattern, while the remaining individual K76069 (*C*. *stygia*; Dunedin, New Zealand) was classified as backcrossed (Figure 4c and Figure S3c, Tables S2 and S3).

## 4 DISCUSSION

In this study, we used genome‐wide SNP data to analyse population structure, genetic connectivity and potential hybridisation of two invasive blowfly species collected from various populations across New Zealand and Australia.

Gene flow is expected to increase fitness and adaptive potential by rapidly spreading beneficial alleles across metapopulations (Lee, 2002; Sexton et al., 2009). Furthermore, repeated episodes of gene flow from the source to invasive populations have been shown to be important for invasion success (Sherpa & Després, 2021). Within New Zealand, we found that *C*. *hilli* and *C*. *stygia* showed little genetic differentiation, indicating high genetic connectivity among populations across the country for both species. Blowflies are highly vagile insects that have been reported to disperse as far as 6 km within 24 h (Norris, 1959), while other flies (e.g. *Stomoxys calcitrans*) have been shown to migrate as far as 225 km (Hogsette & Ruff, 1985). Thus, our findings suggest that the high dispersal capabilities of blowflies, and a general absence of dispersal barriers, have resulted in limited genetic differentiation over large geographical distances. Similar findings have been reported in Australian *Chrysomya* (Butterworth et al., 2023) and North American *Phormia regina* (Picard & Wells, 2009)—though substantial genetic differentiation has been shown in other species (e.g. North American *Lucilia sericata*; Picard & Wells, 2010).

As Australia is the most plausible origin of *C*. *hilli* and *C*. *stygia* into New Zealand, we expected to find strong admixture (i.e. shared genetic ancestries) between both countries, especially considering that their introduction occurred on a very recent evolutionary timescale. However, *C*. *hilli* populations from New Zealand shared very little of their genetic ancestry (<10%) with the Australian cluster, while *C*. *stygia* shared only slightly more (<~20%). These results might be indicative of insufficient Australian sampling and/or founder effects (e.g. genetic bottlenecks that remove alleles from the gene pool and therefore dilute genetic relationships between founding and colonised populations), the latter of which would also explain the low genetic diversity (heterozygosity) observed within each species.

However, we did find high admixture between Australia and New Zealand for a limited number of individuals. For example, all *C*. *hilli* individuals from Blackheath (Australia) shared ~75% of their genetic ancestry with the New Zealand cluster, while two individuals from Jervis Bay and one individual from Mt Keira shared ~40%–75% and ~5% ancestry with New Zealand, respectively. Similarly, several *C*. *stygia* individuals from each of these Australian populations shared genetic ancestry (<50%) with the New Zealand cluster. While this finding reinforces Australia as the source of New Zealand *C*. *hilli* and *C*. *stygia* populations (Dear, 1986), it might also indicate differential sorting of ancestral variation or incomplete lineage sorting as a consequence of genetic drift, causing some populations to be fully differentiated while others retain shared ancestry between Australia and New Zealand (Zhou et al., 2017).

Although our hybrid analyses may have been affected by sample contamination during DNA extraction and/or sequencing, we found compelling evidence—in the form of overlapping samples assigned to opposite species in the PC space for our PCA analysis, admixture in the combined species admixture analysis and identification of hybrid and backcrossed individuals in our hybrid analysis—that interspecific hybridisation is occurring in the wild between *C*. *stygia* and *C*. *hilli*. This is consistent with previous knowledge about the propensity of both species to readily hybridise with other *Calliphora* species under laboratory conditions (Monzu, 1977; Wallman & Adams, 1997), as well as their phylogenetic and ecological relatedness (Dear, 1986; Wallman et al., 2005).

Reproduction between species has been shown to facilitate invasive success in a number of studies (Rius & Darling, 2014; Yamaguchi et al., 2019). For example, hybrid lineages of the apple snails *Pomacea canaliculate* and *Pomacea maculata* in the invasive range in Malaysia acquired traits that significantly enhanced invasiveness via improved desiccation and cold tolerance (Kannan et al., 2021). Meanwhile, hybridisation between the sunflowers *Helianthus annuus* and *Helianthus debilis* (forming the natural hybrid *H*. *annuus* ssp. *texanus*) resulted in hybrid fitness exceeding that of control lines by up to 51% within seven generations in a common garden experiment (Mitchell et al., 2019). However, interspecific hybridisation might also act maladaptively—for example, it can result in outbreeding depression, where the hybrid offspring has reduced fitness compared to its parents (e.g. due to mismatched adaptations or erosion of co‐adapted gene complexes; Muhlfeld et al., 2009). The fact that we identified only a small number of hybrids and late‐generation backcrossed individuals here may suggest that hybridisation in these species is in fact maladaptive, with selection against this process keeping it at low frequencies in the wild. Future work using full‐genome resequencing data and a greater sample size would allow for a broader comparative genomics study which, in conjunction with laboratory‐based fitness assays, could help to differentiate maladaptive versus adaptive hybridisation and, in the latter case, investigate the key genetic traits resulting from hybridisation that may aid invasion in invertebrates.

Overall, our study has provided new insights into the population structure of two invasive blowflies that have crossed the Tasman Sea in replicated incursion events, as well as presented evidence of hybridisation in their respective evolutionary histories. In future, similar studies on other invasive invertebrates—especially those that differ in their degree of invasiveness and time since invasion—will enable broader advances in our understanding of how hybridisation acts in the wild to potentially facilitate invasion. Greater sampling of Australian populations, and the use of demographic modelling analyses, would aid further investigation of the invasion history of these two blowflies, including to more accurately establish their exact pathway and timing of arrival in New Zealand. Such information, alongside the selective use of crosses and their associated fitness effects in laboratory settings, would help to further elucidate the adaptive impacts of hybridisation in biological invasions.

## AUTHOR CONTRIBUTIONS

**Lilly Croft:** Data curation (lead); formal analysis (equal); investigation (equal); methodology (equal); visualisation (equal); writing – original draft (lead); writing – review and editing (supporting). **Paige Matheson:** Data curation (supporting); formal analysis (equal); investigation (equal); methodology (equal); visualisation (equal); writing – original draft (supporting); writing – review and editing (lead). **Chloe Flemming:** Data curation (supporting); methodology (supporting); writing – review and editing (supporting). **Nathan J. Butterworth:** Supervision (supporting); writing – review and editing (supporting). **Angela McGaughran:** Conceptualisation (lead); formal analysis (supporting); funding acquisition (lead); investigation (supporting); methodology (supporting); project administration (lead); resources (lead); supervision (lead); validation (supporting); visualisation (supporting); writing – original draft (supporting); writing – review and editing (supporting).

## FUNDING INFORMATION

This work was funded by Te Whare Wānanga o Waikato/The University of Waikato (Research Support Grant to AM; Summer Research Scholarship to AM to support CF).

## CONFLICT OF INTEREST STATEMENT

The authors of this article declare that they have no conflict of interest.

## BENEFIT‐SHARING STATEMENT

Benefits from this research accrue from the sharing of our data as Supporting Information, as described above.

## Supporting information


Appendix S1.

## Data Availability

The raw sequence data are deposited in the NCBI Sequence Read Archive (SRA) database, BioProject ID: PRJNA1052751; associated metadata is provided in Table S1.
