# Supplementary material for: Population structure and interspecific hybridisation of two invasive blowflies (Diptera: Calliphoridae) following replicated incursions into New Zealand
Source: Ecol Evol. 2024 Jan 7;14(1):e10832. doi: 10.1002/ece3.10832 (PMC10772223; doi:10.1002/ece3.10832)
Supplement: Supplementary file 1 — Appendix S1. [file ECE3-14-e10832-s001.docx]

**Supplementary Information**

**Population structure and interspecific hybridisation of two invasive blowflies (Diptera: Calliphoridae) following replicated incursions to New Zealand**

Lilly Croft^1^, Paige Matheson^1^, Chloe Flemming^1^, Nathan J. Butterworth^2^, Angela McGaughran*^1^

^1^Te Aka Mātuatua - School of Science, University of Waikato, Private Bag 3105, Hamilton 3240, New Zealand

^2^School of Biological Sciences, Monash University, Clayton, Victoria 3800, Australia

**Figure S1**. PCA plots based on non-neutral datasets for Calliphora hilli (18,324 SNPs), coloured by region (A) and population (B); and for Calliphora stygia (16,115 SNPs), by region (C) and population (D). These plots are generally consistent with the ones generated for the neutral dataset (Fig. 2A,B).

**(A)**


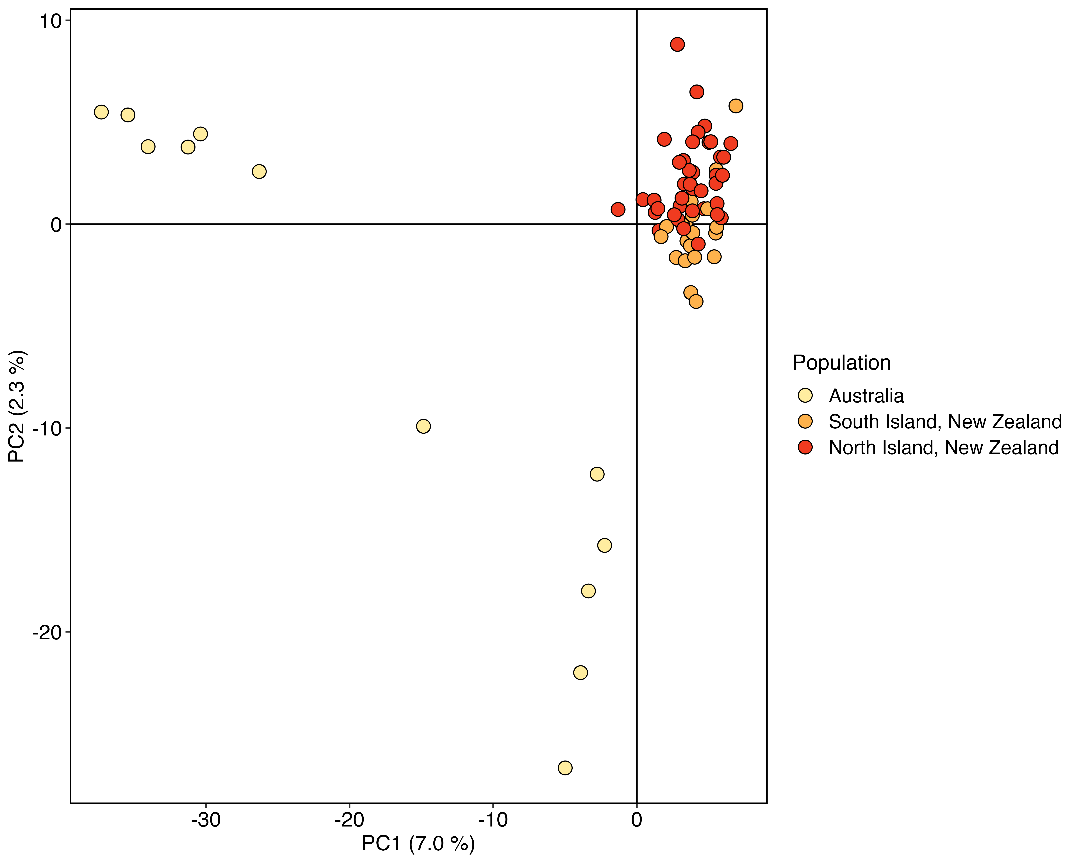


**(B)**


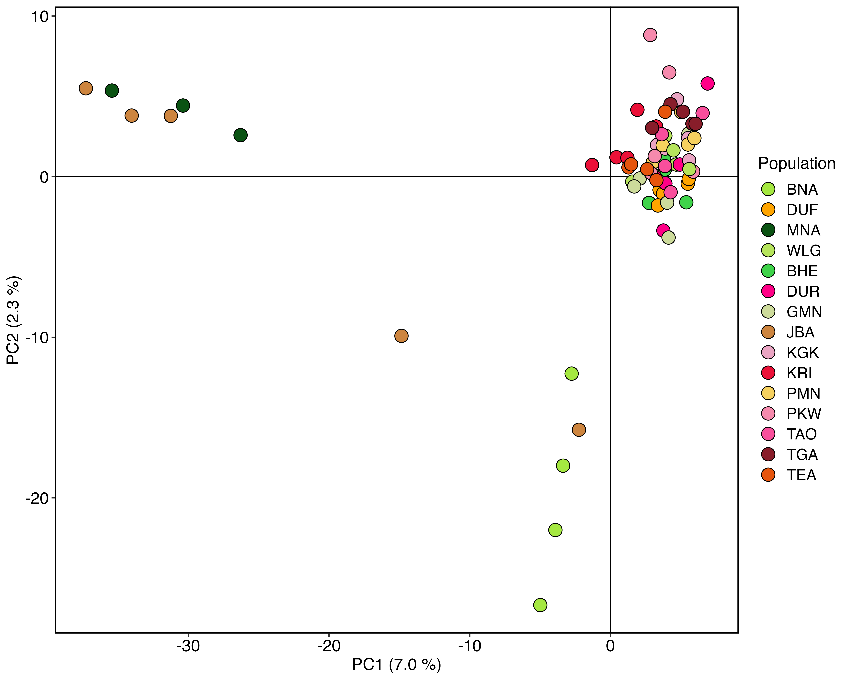


**(C)**


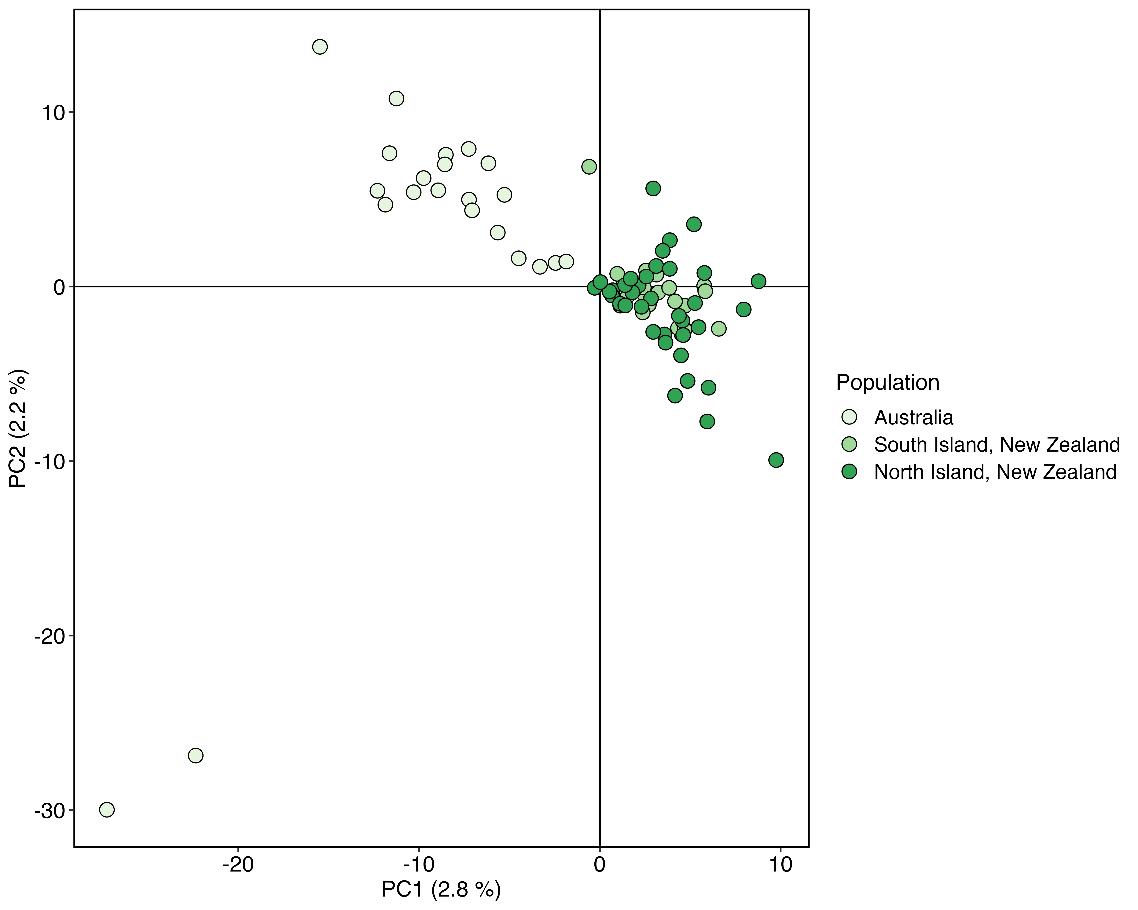


**(D)**


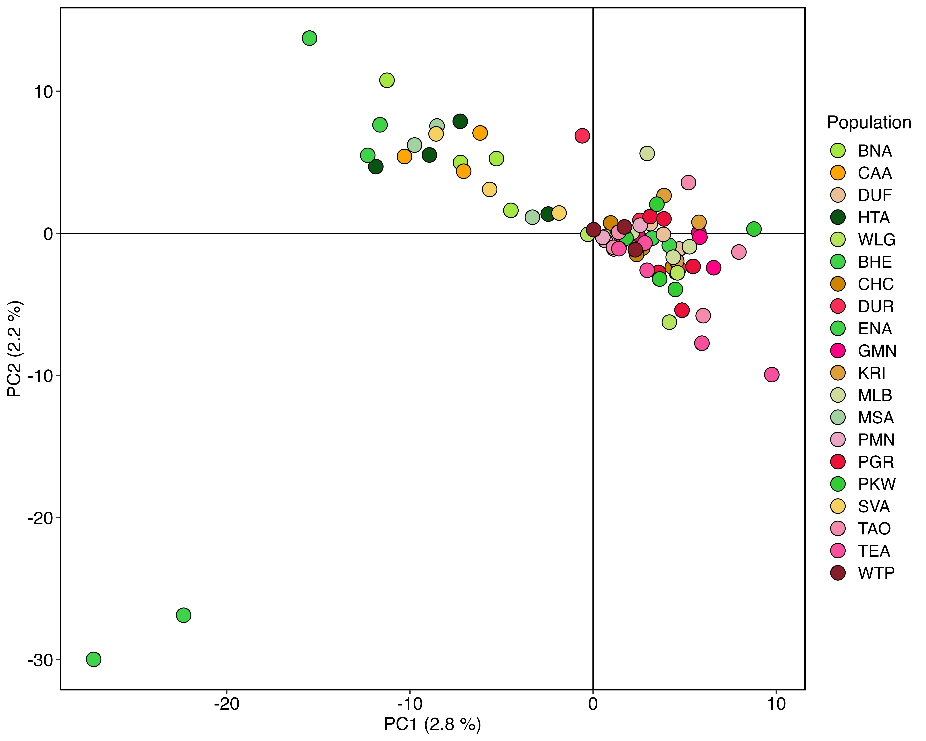


**Figure S2**. Admixture plots for non-neutral datasets for: (A) *Calliphora hilli* (16,144 SNPs; and (B) *Calliphora stygia* (16,115 SNPs). Plots were produced using Sparse Non-Negative Matrix Factorisation (sNMF) analysis, with the optimal K value determined to be K=2 for both species and admixture proportions presented for K=2 to K=5. Results are consistent with the neutral dataset for both species (Fig. 3A,B).

**(A)**


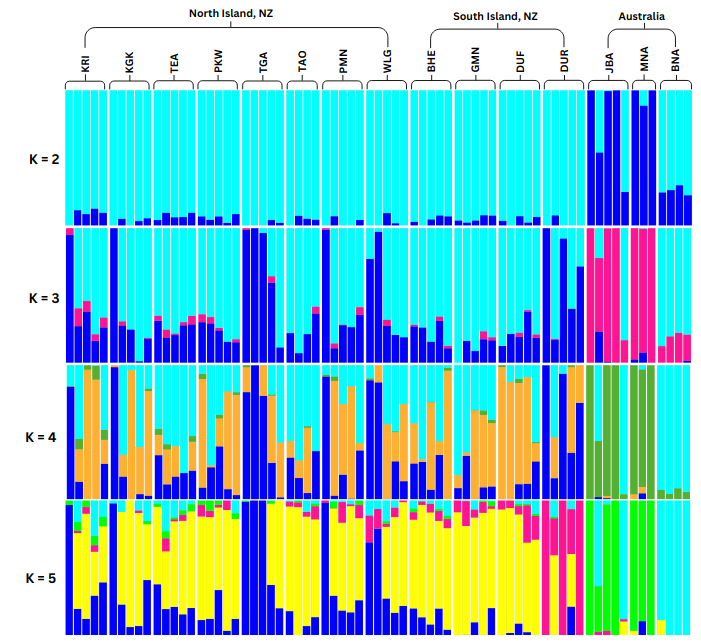


**(B)**


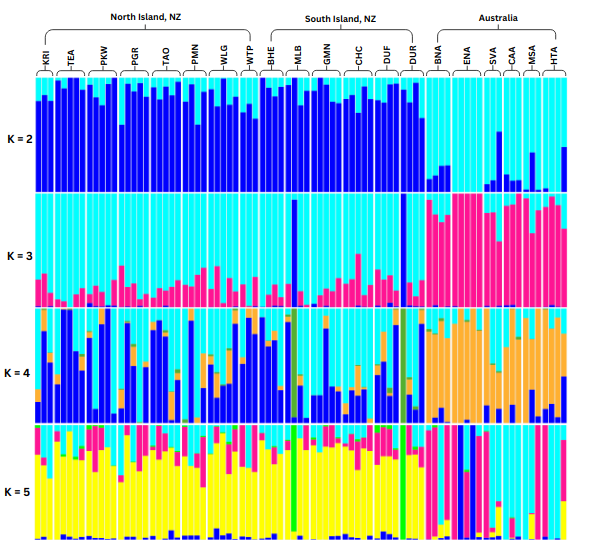


**Figure S3**. Results from hybrid analysis of the combined *Calliphora hilli* and *Calliphora stygia* samples from the non-neutral dataset (16,333 SNPs). (A) PCA plot, with potential hybrids labelled by individual codes and individuals coloured by their taxonomically identified species; (B) Admixture plot for K-values of 2-5, produced using Sparse Non-Negative Matrix Factorisation (sNMF) analysis; and (C) NewHybrids analysis, indicating ‘pure’, hybrid, or backcrossed status of 13 individuals. F1 and F2 hybrids represent offspring from first and second generation crosses between *C. stygia* and *C. hilli*, respectively. BC to *C. hilli* and BC to *C. stygia* indicates first generation back-crossed individuals to the respective species. Each individual is labeled according to its taxonomic identification, with individual codes corresponding to Table S1. Results are highly consistent with the neutral dataset (Fig. 4).

**(A)**


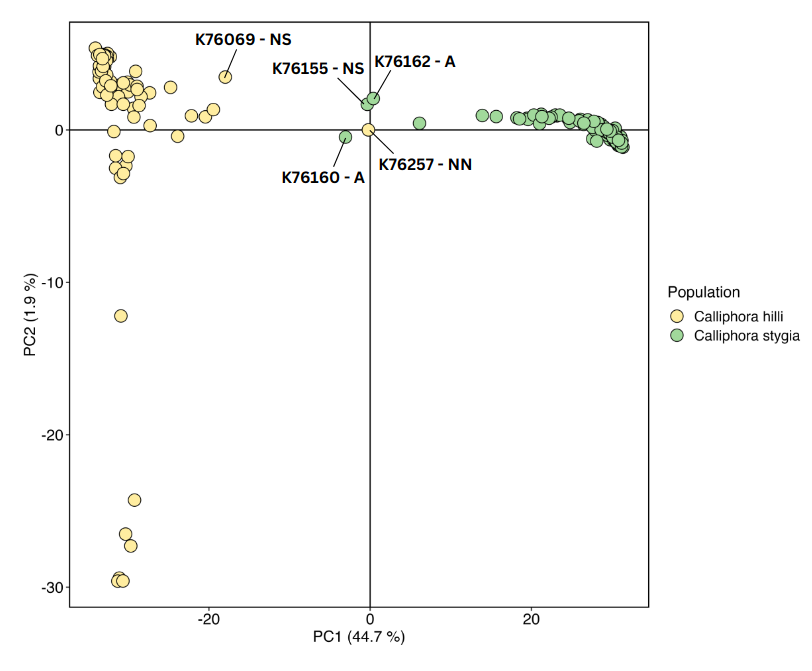


**(B)**


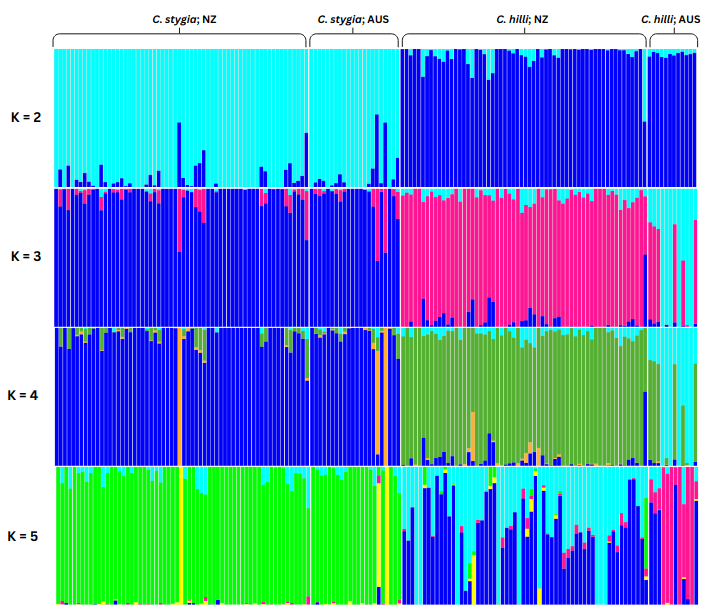


**(C)**


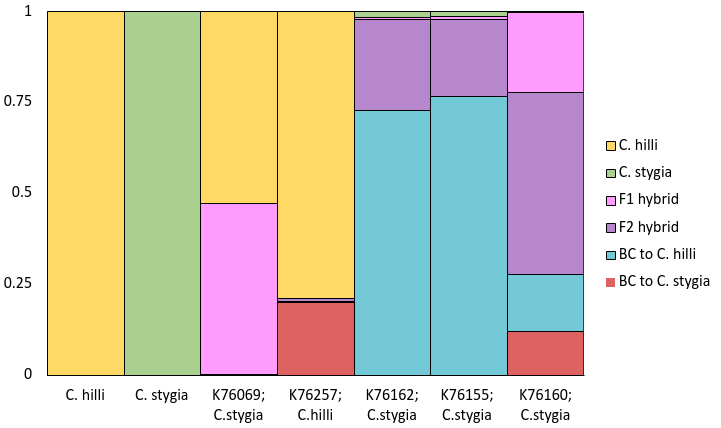


**Table S1.** Individual sampling information for *Calliphora hilli* and *Calliphora stygia*, including population names, specimen identification codes, and dates of collection (unknown dates are left blank or indicated with ‘x’). New Zealand populations are listed in rough geographical order from the top of the North Island to the bottom of the South Island. Samples highlighted in blue represent individuals that were removed from the dataset prior to population analyses due to the results of the kinship analysis; samples highlighted in orange were also removed due to the population having a low sample size following the removal of related individuals.

| **Location** | **Sample ID** | **Sample collection date** | **Species** |
| --- | --- | --- | --- |
| KRI | K76149 | xx.11.20 | *C. stygia* |
| KRI | K76150 | xx.11.20 | *C. stygia* |
| KRI | K76151 | xx.11.20 | *C. stygia* |
| KRI | K76152 | xx.11.20 | *C. stygia* |
| KRI | K76144 | xx.11.20 | *C. hilli* |
| KRI | K76145 | xx.11.20 | *C. hilli* |
| KRI | K76146 | xx.11.20 | *C. hilli* |
| KRI | K76147 | xx.11.20 | *C. hilli* |
| KRI | K76148 | xx.11.20 | *C. hilli* |
| KGK | K76132 | 18.12.21 | *C. hilli* |
| KGK | K76133 | 18.12.21 | *C. hilli* |
| KGK | K76134 | 18.12.21 | *C. hilli* |
| KGK | K76135 | 18.12.21 | *C. hilli* |
| KGK | K76136 | 18.12.21 | *C. hilli* |
| TEA | K76240 | 29.01.22 | *C. stygia* |
| TEA | K76241 | 29.01.22 | *C. stygia* |
| TEA | K76242 | 29.01.22 | *C. stygia* |
| TEA | K76243 | 29.01.22 | *C. stygia* |
| TEA | K76244 | 29.01.22 | *C. stygia* |
| TEA | K76233 | 29.01.22 | *C. hilli* |
| TEA | K76234 | 29.01.22 | *C. hilli* |
| TEA | K76235 | 29.01.22 | *C. hilli* |
| TEA | K76236 | 29.01.22 | *C. hilli* |
| TEA | K76237 | 29.01.22 | *C. hilli* |
| PKW | K76196 | 10.12.21 | *C. stygia* |
| PKW | K76197 | 10.12.21 | *C. stygia* |
| PKW | K76198 | 10.12.21 | *C. stygia* |
| PKW | K76199 | 10.12.21 | *C. stygia* |
| PKW | K76190 | 10.12.21 | *C. hilli* |
| PKW | K76191 | 10.12.21 | *C. hilli* |
| PKW | K76192 | 10.12.21 | *C. hilli* |
| PKW | K76193 | 10.12.21 | *C. hilli* |
| PKW | K76194 | 10.12.21 | *C. hilli* |
| PGR | K76183 | 18.01.22 | *C. stygia* |
| PGR | K76184 | 18.01.22 | *C. stygia* |
| PGR | K76185 | 18.01.22 | *C. stygia* |
| PGR | K76186 | 18.01.22 | *C. stygia* |
| PGR | K76187 | 18.01.22 | *C. stygia* |
| TGA | K76223 | 01.09.20 | *C. hilli* |
| TGA | K76224 | 01.09.20 | *C. hilli* |
| TGA | K76225 | 01.09.20 | *C. hilli* |
| TGA | K76226 | 01.09.20 | *C. hilli* |
| TGA | K76227 | 01.09.20 | *C. hilli* |
| TAO | K76213 |  | *C. stygia* |
| TAO | K76214 |  | *C. stygia* |
| TAO | K76215 |  | *C. stygia* |
| TAO | K76216 |  | *C. stygia* |
| TAO | K76217 |  | *C. stygia* |
| TAO | K76208 |  | *C. hilli* |
| TAO | K76209 |  | *C. hilli* |
| TAO | K76211 |  | *C. hilli* |
| TAO | K76212 |  | *C. hilli* |
| PMN | K76168 | 09.11.21 | *C. stygia* |
| PMN | K76169 | 09.11.21 | *C. stygia* |
| PMN | K76170 | 09.11.21 | *C. stygia* |
| PMN | K76171 | 09.11.21 | *C. stygia* |
| PMN | K76172 | 09.11.21 | *C. stygia* |
| PMN | K76163 | 09.11.21 | *C. hilli* |
| PMN | K76164 | 09.11.21 | *C. hilli* |
| PMN | K76165 | 09.11.21 | *C. hilli* |
| PMN | K76166 | 09.11.21 | *C. hilli* |
| PMN | K76167 | 09.11.21 | *C. hilli* |
| WLG | K76019 | 09.02.21 | *C. stygia* |
| WLG | K76020 | 09.02.21 | *C. stygia* |
| WLG | K76021 | 09.02.21 | *C. stygia* |
| WLG | K76249 | 09.02.21 | *C. stygia* |
| WLG | K76250 | 09.02.21 | *C. stygia* |
| WLG | K76016 | 09.02.21 | *C. hilli* |
| WLG | K76017 | 09.02.21 | *C. hilli* |
| WLG | K76018 | 09.02.21 | *C. hilli* |
| WLG | K76247 | 09.02.21 | *C. hilli* |
| WLG | K76248 | 09.02.21 | *C. hilli* |
| WTP | K76259 | 10.02.18 | *C. stygia* |
| WTP | K76260 | 10.02.18 | *C. stygia* |
| WTP | K76261 | 14.11.12 | *C. stygia* |
| HYT | K76114 | 26.01.21 | *C. hilli* |
| HYT | K76115 | 26.01.21 | *C. hilli* |
| HYT | K76116 | 26.01.21 | *C. hilli* |
| HYT | K76117 | 26.01.21 | *C. hilli* |
| BHE | K76033 | 12.01.21 | *C. stygia* |
| BHE | K76034 | 12.01.21 | *C. stygia* |
| BHE | K76035 | 12.01.21 | *C. stygia* |
| BHE | K76036 | 12.01.21 | *C. stygia* |
| BHE | K76027 | 12.01.21 | *C. hilli* |
| BHE | K76028 | 12.01.21 | *C. hilli* |
| BHE | K76029 | 12.01.21 | *C. hilli* |
| BHE | K76030 | 12.01.21 | *C. hilli* |
| BHE | K76031 | 12.01.21 | *C. hilli* |
| MLB | K76155 | 12.01.21 | *C. stygia* |
| MLB | K76156 | 12.01.21 | *C. stygia* |
| MLB | K76157 | 12.01.21 | *C. stygia* |
| GMN | K76097 | 16.06.21 | *C. stygia* |
| GMN | K76098 | 16.06.21 | *C. stygia* |
| GMN | K76099 | 16.06.21 | *C. stygia* |
| GMN | K76100 | 16.06.21 | *C. stygia* |
| GMN | K76101 | 16.06.21 | *C. stygia* |
| GMN | K76087 | 16.06.21 | *C. hilli* |
| GMN | K76088 | 16.06.21 | *C. hilli* |
| GMN | K76089 | 16.06.21 | *C. hilli* |
| GMN | K76090 | 16.06.21 | *C. hilli* |
| GMN | K76091 | 16.06.21 | *C. hilli* |
| CHC | K76044 | 13.11.20 | *C. stygia* |
| CHC | K76045 | 13.11.20 | *C. stygia* |
| CHC | K76046 | 13.11.20 | *C. stygia* |
| CHC | K76047 | 13.11.20 | *C. stygia* |
| CHC | K76048 | 13.11.20 | *C. stygia* |
| DUF | K76004 | 08.06.21 | *C. stygia* |
| DUF | K76005 | 08.06.21 | *C. stygia* |
| DUF | K76006 | 08.06.21 | *C. stygia* |
| DUF | K76058 | 08.06.21 | *C. stygia* |
| DUF | K76059 | 08.06.21 | *C. stygia* |
| DUF | K76001 | 08.06.21 | *C. hilli* |
| DUF | K76002 | 08.06.21 | *C. hilli* |
| DUF | K76003 | 08.06.21 | *C. hilli* |
| DUF | K76054 | 08.06.21 | *C. hilli* |
| DUF | K76055 | 08.06.21 | *C. hilli* |
| DUR | K76070 | 24.05.21 | *C. stygia* |
| DUR | K76071 | 24.05.21 | *C. stygia* |
| DUR | K76072 | 24.05.21 | *C. stygia* |
| DUR | K76073 | 24.05.21 | *C. stygia* |
| DUR | K76065 | 24.05.21 | *C. hilli* |
| DUR | K76066 | 24.05.21 | *C. hilli* |
| DUR | K76067 | 24.05.21 | *C. hilli* |
| DUR | K76068 | 24.05.21 | *C. hilli* |
| DUR | K76069 | 24.05.21 | *C. hilli* |
| JBA | K76127 |  | *C. hilli* |
| JBA | K76128 |  | *C. hilli* |
| JBA | K76129 |  | *C. hilli* |
| JBA | K76130 |  | *C. hilli* |
| JBA | K76131 |  | *C. hilli* |
| MNA | K76010 | xx.02.05 | *C. hilli* |
| MNA | K76011 | xx.02.05 | *C. hilli* |
| MNA | K76012 | xx.02.05 | *C. hilli* |
| BNA | K75995 | 24.04.21 | *C. stygia* |
| BNA | K75996 | 24.04.21 | *C. stygia* |
| BNA | K75997 | 24.04.21 | *C. stygia* |
| BNA | K76025 | 24.04.21 | *C. stygia* |
| BNA | K76026 | 24.04.21 | *C. stygia* |
| BNA | K75992 | 24.04.21 | *C. hilli* |
| BNA | K75993 | 24.04.21 | *C. hilli* |
| BNA | K75994 | 24.04.21 | *C. hilli* |
| BNA | K76024 | 24.04.21 | *C. hilli* |
| ENA | K76078 | 03.06.08 | *C. stygia* |
| ENA | K76079 | 03.06.08 | *C. stygia* |
| ENA | K76080 | 03.06.08 | *C. stygia* |
| ENA | K76081 | 03.06.08 | *C. stygia* |
| ENA | K76082 | 03.06.08 | *C. stygia* |
| SVA | K76205 | 10.05.01 | *C. stygia* |
| SVA | K76206 | 10.05.01 | *C. stygia* |
| SVA | K76207 | 10.05.01 | *C. stygia* |
| CAA | K75998 | 13.10.04 | *C. stygia* |
| CAA | K75999 | 13.10.05 | *C. stygia* |
| CAA | K76000 | 13.10.04 | *C. stygia* |
| CAA | K76043 | 13.10.04 | *C. stygia* |
| MSA | K76158 |  | *C. stygia* |
| MSA | K76159 |  | *C. stygia* |
| MSA | K76161 |  | *C. stygia* |
| MSA | K76162 |  | *C. stygia* |
| HTA | K76008 | 30.09.03 | *C. stygia* |
| HTA | K76009 | 30.09.03 | *C. stygia* |
| HTA | K76120 | 30.09.03 | *C. stygia* |
| HTA | K76121 | 30.09.03 | *C. stygia* |

**Table S3.** Hybrid results averaged from ten rounds of NewHybrids analysis of the neutral dataset using the ‘random’ method. In the table, ‘pure’, F1/F2 hybrid, or backcrossed status of six individuals are presented, using a threshold of 0.01*.* F1 and F2 hybrids represent offspring from first- and second-generation crosses between *C. stygia* and *C. hilli*, respectively*,* BC to *C. hilli* and BC to *C. stygia* indicates first generation back-crossed individuals to the respective species*,* each individual is labeled in the first column according to its taxonomic identification, with individual codes corresponding to Table S1.

| **Individual** | **Species** | ***C. stygia*** | ***C. hilli*** | **F1 hybrid** | **F2 hybrid** | **BC to *C. stygia*** | **BC to *C. hilli*** |
| --- | --- | --- | --- | --- | --- | --- | --- |
| C. hilli | *C. hilli* | 0 | 1 | 0 | 0 | 0 | 0 |
| C. stygia | *C. stygia* | 1 | 0 | 0 | 0 | 0 | 0 |
| K76069; *C. stygia* | *C. hilli* | 0 | 0.846 | 0 | 0.000085 | 0.154164 | 0 |
| K76257; *C. hilli* | *C. hilli* | 0.242462 | 0.754623 | 0.002371 | 0.000823 | 0.001107 | 0.000506 |
| K76162; *C. stygia* | *C. stygia* | 0.014387 | 0.000003 | 0.017103 | 0.200630 | 0 | 0.767876 |
| K76155; *C. stygia* | *C. stygia* | 0.005461 | 0 | 0.036079 | 0.301203 | 0.000137 | 0.65712 |
| K76160; *C. stygia* | *C. stygia* | 0.000010 | 0.001587 | 0.291334 | 0.392699 | 0.179647 | 0.134724 |

**Table S4.** Hybrid results averaged from ten rounds of NewHybrids analysis of the non-neutral dataset using the ‘random’ method. In the table, ‘pure’, F1/F2 hybrid, or backcrossed status of six individuals are presented, using a threshold of 0.01*.* F1 and F2 hybrids represent offspring from first- and second-generation crosses between *C. stygia* and *C. hilli*, respectively*,* BC to *C. hilli* and BC to *C. stygia* indicates first generation back-crossed individuals to the respective species*,* each individual is labeled in the first column according to its taxonomic identification, with individual codes corresponding to Table S1.

| **Individual** | **Species** | **BC to *C. stygia*** | **BC to *C. hilli*** | **F2 hybrid** | **F1 hybrid** | ***C. stygia*** | ***C. hilli*** |
| --- | --- | --- | --- | --- | --- | --- | --- |
| *C. hilli* | *C. hilli* | 0 | 0 | 0 | 0 | 0 | 1 |
| *C. stygia* | *C. stygia* | 0 | 0 | 0 | 0 | 1 | 0 |
| K76069; *C. stygia* | *C. hilli* | 0.001135 | 0.000964 | 0.001087 | 0.469985 | 0.000949 | 0.525881 |
| K76257; *C. hilli* | *C. hilli* | 0.201911 | 0.000506 | 0.00912 | 0 | 0 | 0.78897 |
| K76162; *C. stygia* | *C. stygia* | 0 | 0.728505 | 0.249831 | 0.005672 | 0.015956 | 0.000035 |
| K76155; *C. stygia* | *C. stygia* | 0.000002 | 0.767115 | 0.2113 | 0.009914 | 0.011668 | 0 |
| K76160; *C. stygia* | *C. stygia* | 0.12227 | 0.155171 | 0.500678 | 0.220449 | 0.001284 | 0.000148 |
